# State-of-the-Art in Biosafety and Biosecurity in European Countries

**DOI:** 10.1007/s00005-014-0290-1

**Published:** 2014-05-13

**Authors:** Anna Bielecka, Ali Akbar Mohammadi

**Affiliations:** 1 Military Institute of Hygiene and Epidemiology, Warsaw, Poland; 2 Global Health and Security Consultants, Geneva, Switzerland

**Keywords:** Biosafety, Biosecurity, Legislation, BTWC

## Abstract

The terms biosafety and biosecurity are widely used in different concepts and refer not only to protection of human beings and their surrounding environment against hazardous biological agent, but also to global disarmament of weapons of mass destruction. As a result, the biosafety and biosecurity issues should be considered interdisciplinary based on multilateral agreements against proliferation of biological weapons, public health and environmental protection. This publication presents information on both, international and national biosafety and biosecurity legislation. Status of national implementation of the Biological and Toxin Weapons Convention, penalization issues and measures to account for and secure production, use, storage of particularly dangerous pathogens or activities involving humans, plants and animals where infection may pose a risk have been analyzed. Safety and security measures in laboratories have been studied. Moreover, dual-use technology and measures of secure transport of biohazard materials have been also taken into account. In addition, genetic engineering regulations, biosecurity activities in laboratories and code of conducts have been investigated, as well.

## Introduction

The terms biosafety and biosecurity are widely used in different concepts and frameworks which basically include best practices in working safely and securely with biological agents and toxins in different workplaces and environments.

However, biosafety and biosecurity initiatives refer not only to protection of human beings and their surrounding environment against hazardous biological agent, but also to global disarmament of weapons of mass destruction. As a result, the biosafety and biosecurity issues need to be considered interdisciplinary based on multilateral agreements against proliferation of biological weapons, public health and environmental protection.

The Biological and Toxin Weapons Convention (BTWC) is a key element in the international community’s efforts to address the proliferation of weapons of mass destruction. The main provisions of the BTWC cover prohibitions, implementation, protection and peaceful usage of biological and toxin weapons (Millett 2010). The Convention was opened for signature on 10 April 1972 and entered into force on 26 March 1975. Currently, there are 170 States Parties, which signed and ratified the BTWC, another 10 states have signed, but not ratified the treaty and 16 states are not members (UNOG The United Nations Office at Geneva, Membership of Biological Weapons Convention 2013).

In accordance with article IV of the BTWC, each State Party shall, in agreement with its constitutional ordinance, take activities to prohibit and prevent the development, production, stockpiling, acquisition of biological agents, toxins, weapons, equipment and means of delivery. In addition, article III requires all States Parties to refrain from transferring biological weapons to anyone and from assisting, encouraging or including anyone to produce or acquire them. Based on this, some issues should be considered in a particular way, namely, penal measures, biosafety and biosecurity measures, import and export controls, enforcement measures and both, domestic and international cooperation and assistance.

It is still difficult to balance efforts to improve public health with efforts to counter bioterrorism despite slight/subtle differences between natural and deliberate outbreaks of diseases (Dembek et al. 2007; Hunger et al. 2013). Moreover, clinical symptoms caused by biological agents of public health concern may mimic some agents of bioterrorism concern and vice versa. To assist State Parties in the implementation of the article X of BTWC, an International Health Regulation (IHR) has been issued by the World Health Organization (WHO). The IHR supports the State Parties in capability building to prepare and respond to natural, accidental and intentional spread of diseases as well as to improve the Confidence Building Measures information exchange process. By adoption of IHR, States Parties become more aware of the connection between public health and biological weapons reflecting in closer integration of WHO and BTWC activities on biosafety, biosecurity, disease surveillance and reporting.

Finally, environmental protection needs to be in biological safety and security concern, as well. Modern biotechnology, including synthetic biology, -nomics sciences, is an emerging instrument with potentials in improving human and animal health, agriculture, industrial and agricultural production as well as environmental protection. Nevertheless, the development and applications of it may have potential side effects to human and animal health as well as environment, counting biodiversity risks. Regulation of modern biotechnology products ought to safeguard the right of consumers to have access to safe products, information about the products and live in a safe and sustainable environment. The Cartagena Protocol on Biosafety has established a legal framework for international trade in genetically modified organism (GMOs) and provides Signatory State Parties with orientation and the framework for development of complementary national biosafety regulations. It however, does not address the risks and safe practices of handling of such organisms in different workplaces.

As a result, State Parties were obligated to review their domestic legislation and/or implement an additional law(s). National implementation of BTWC includes not only legislative and administrative measures, but also actions to enhance native compliance with the BTWC, export control system, biological safety and security, disease surveillance, biological agents detection, education and awareness raising.

This publication presents information on both, international and national biosafety and biosecurity legislation. Status of national implementation of BTWC, penalization issues and measures to account for and secure production, use, storage of particularly dangerous pathogens or activities involving humans, plants and animals where infection may pose a risk have been analyzed. Safety and security measures in laboratories including laboratory safety equipment, personnel protective equipment, waste handling and accident reporting have been studied. Moreover, dual-use technology and measures of secure transport of biohazard materials have been also taken into account. In addition, genetic engineering regulations, biosecurity activities in laboratories and code of conducts have been investigated, as well.

## European Union Regulations in Biosafety and Biosecurity

The second, third and fourth Review Conferences noted “the importance of … legislation regarding the physical protection of laboratories and facilities to prevent unauthorized access to and removal of microbial or other biological agents, or toxins” (Final Declaration of the Fourth Review Conference 1996a; Final Declaration of the Third Review Conference 1991a; Final Declaration of the Second Review Conference 1986a). Following, the Sixth Review Conference recognized “that states must take all necessary safety and security provisions to protect populations and the environment when carrying out such destruction and/or diversion” (Final Declaration of the Sixth Review Conference 2006a). Moreover, the Sixth Review Conference called “upon States Parties to adopt, in accordance with their constitutional processes, legislative, administrative, judicial and other measures, including penal legislation, designed to … ensure the safety and security of microbial or other biological agents or toxins in laboratories, facilities, and during transportation, to prevent unauthorized access to and removal of such agents and toxins” (Final Declaration of the Sixth Review Conference 2006b).

Final Declaration of the Seventh Review Conference calls for “need to implement safety and security measures to protect human populations and the environment, including animals and plants” in accordance to article II (Final Declaration of the Seventh Review Conference 2012a), “need to ensure safety and security of microbial or other biological agents or toxins in laboratories, facilities, and during transportation, to prevent unauthorized access to and removal of such agents and toxins” in accordance to article IV (Final Declaration of the Seventh Review Conference 2012b). as well as “need to national implementation measures” regarding standards on biosafety and biosecurity, awareness among relevant professionals in public sector and sciences, promulgation of codes of conduct, training and education programs, capability building for surveillance and detection of outbreaks (IHR), equipment and means of delivery control of transport in accordance to paragraph 13 of article IV (Final Declaration of the Seventh Review Conference 2012b). In reference to disease surveillance and detection, the Sixth Review Conference has previously reaffirmed that “the commitment of State Parties to take the necessary national measures to strengthen methods and capabilities for surveillance and detection of outbreaks of disease at the national, regional and international levels” (Final Declaration of the Sixth Review Conference 2006c).

Referring to public, human and animal health the second, third and fourth Review Conferences called “for greater cooperation in international public health and disease control” (Final Declaration of the Second Review Conference 1986b; Final Declaration of the Third Review Conference 1991b; Final Declaration of the Fourth Review Conference 1996b). The third and fourth Review Conferences additionally urged “in providing information on national epidemiological surveillance and data reporting systems, and (…) assistance, on a bilateral level and/or in conjunction with WHO, the Food and Agriculture Organization (FAO) of the United Nations and the World Organization for Animal Health (OIE), regarding epidemiological and epizootical surveillance, with a review to improvements in the identification and timely reporting of significant outbreaks of human and animal diseases” (Final Declaration of the Third Review Conference 1991b; Final Declaration of the Fourth Review Conference 1996b). The Sixth Review Conference broadens above area calling for disease surveillance specific for humans, animals and plants (Final Declaration of the Sixth Review Conference 2006e), improvement of “communication on disease surveillance at all levels” (Final Declaration of the Sixth Review Conference 2006f) and “improving national and regional capabilities to survey, detect, diagnose and combat infectious diseases as well as other possible biological threats and integrate these efforts into national and/or regional emergency and disease management plans” (Final Declaration of the Sixth Review Conference 2006f). Moreover, it also calls for promotion of development and production of vaccines and drugs used in infectious diseases treatment (Final Declaration of the Sixth Review Conference 2006d, f).

The European Union Member States in parallel with the BTWC process have also collectively and individually established effective regulations towards implementation of biosafety and biosecurity.

The list of currently applicable laws and regulations related to biosafety and biosecurity in European Union (EU) is shown in Table 1.

**Table 1:** European Union (EU) regulations related to biosafety and biosecurity sectors

Name	Objectives
Directive 89/391/EEC, 12 June 1989	Introduces measures to encourage improvements in the safety and health of workers at work
Directive 89/656/EEC, 30 November 1989	Provides minimum health and safety requirements for the use by workers of personal protective equipment at the workplace
Council directive 90/220/EEC, 3 April 1990	On the deliberate release into the environment of GMOs
Council directive 90/219/EEC, 23 April 1990	On the contained use of GMMs
Council directive 90/679/EEC, 26 November 1990	On the protection of workers from risks related to exposure to biological agents at work. Requires manufacturers, importers, distributors and suppliers to provide safety data sheets for microorganisms (Directive 90/679/EEC has been repealed in September 2000 by Directive 2000/54/EC)
Council regulation 2309/93/EEC, 26 July 1993	Lays down community procedures for the authorization and supervision of medicinal products for human and veterinary use and establishing a European Agency for the Evaluation of Medicinal Products
Council directive 93/88/EEC, 12 October 1993	Amends Council Directive 90/679/EEC on the protection of workers from risks related to exposure to biological agents at work to technical progress
Council directive 93/75/EC, 13 September 1993	Establishes minimum requirements for vessels bound for or leaving Community ports and carrying dangerous or polluting goods
Commission directive 94/51/EEC, 7 November 1994	Amends council directive 90/219/EEC to technical progress on the contained use of GMOs
Commission directive 94/3/EC, 21 January 1994	Establishes procedures for the notification of interception of a consignment or a harmful organism from third countries and presenting an imminent phytosanitary danger
Commission decision 94/730/EEC, 4 November 1994	Establishes simplified procedures on the deliberate release of genetically modified plants into the environment
Council directive 94/55/EC, 21 November 1994	On the approximation of the laws of the Member States on the transport of dangerous goods by road
Council decision 94/942 CFSP, 19 December 1994	On the joint action adopted by the Council on the basis of Article J 3 of the Treaty on EU on export controls of dual use of goods
Council regulation 94/3381/EC, 19 December 1994	On a community regime for the control of exports of dual-use items and technology
Council directive 95/44/EC, 26 July 1995	Establishes the conditions under which certain harmful organisms, plants, plant products and other objects listed in Annexes I to V of Council Directive 77/93/EEC may be introduced into or moved within the Community for trial or scientific purposes
Council directive 95/44/EC, 25 July 1995	Establishes the conditions under which certain harmful organisms, plants, plant products and other objects listed in Annexes I to V to Council Directive 77/93/EEC may be introduced into or moved within the Community or certain protected zones thereof, for trial or scientific purposes and for work on varietal selections
Commission directive 95/30/EC, 30 June 1995	Adapts council directive 90/679/EEC on the protection of workers from risks related to exposure to biological agents at work to technical progress
Council directive 96/49/EC, 23 July 1996	On the approximation of laws of the Member States with regard to the transport of dangerous goods by rail
Council common position 96/408/CFSP, 25 June 1996	Relates to preparation for the Fourth Review Conference of the Convention on the prohibition of the development, production and stockpiling of bacteriological (biological) and toxin weapons and on their destruction (BTWC)
Council decision 96/613/CFSP, 22 October 1996	Amends decision 94/942/CFSP on the joint action adopted by the Council on the control of exports of dual-use goods. Contains the list of dual-use goods subject to controls within the EU
Commission directive 96/87/EC, 13 December 1996	Adapts to technical progress Council Directive 96/49/EC on the approximation of the laws of the Member States with regard to the transport of dangerous goods by rail
Commission directive 96/39/EC, 19 June 1996	Amends council directive 93/75/EEC concerning minimum requirements for vessels bound for or leaving Community ports and carrying dangerous or polluting goods
Council directive 97/35/EC	Amends Council directive 90/220/EC on deliberate release into the environment of GMOs
Commission directive 97/59/EC, 7 October 1997	Adapts Council directive 90/679/EEC on the protection of workers from risks related to exposure to biological agents at work to technical progress
Commission directive 97/65/EC, 26 November 1997	Adapts Council directive 90/679/EEC on the protection of workers from risks related to exposure to biological agents at work to technical progress
Commission directive 97/34/EC, 6 June 1997	Amends Council directive 93/75/EEC concerning minimum requirements for vessels bound for or leaving Community ports and carrying dangerous or polluting goods
Commission directive 97/35/EC of 18 June 1997	Updating Council directive 90/220/EEC on the deliberate release into the environment of GMO’s
Council directive 98/55/EC, 17 July 1998	Amends Council directive 93/75EEC concerning minimum requirements for vessels bound for or leaving Community ports and carrying dangerous or polluting goods
Commission directive 98/74/EC, 1 October 1998	Amends Council directive 93/75EEC concerning minimum requirements for vessels bound for or leaving Community ports and carrying dangerous or polluting goods
Council common position 98/197/CFSP, 4 March 1998	Promotes the early and successful conclusions of Ad Hoc Group negotiations and requires member States to continue to promote universality of the Convention
Council directive 98/81/EC, 26 October 1998	Amends directive 90/219/EEC on the contained use of GMOs. Requires Member States to take all appropriate measures to avoid adverse effects of GMOs and carry out a risk assessment before release into the environment
Council joint action 1999/878/CFSP, 17 December 1999	Establishes a cooperative program for non-proliferation and disarmament in the Russian Federation
Council common position 1999/346/CFSP, 17 May 1999	Relates to progress towards a legally binding Protocol to strengthen compliance with the BTWC and with a view to the successful completion of substantive work in the Ad Hoc Group by the end of 1999
Council directive 2000/54/EC, 18 September 2000	On the protection of workers from risks related to exposure to biological agents at work
Council directive 2000/29/EC, 8 May 2000	On protective measures against the introduction into the Community of organisms harmful to plants or plant products and against their spread within the Community
Council regulation no. 1334/2000	Article 4 extends to items which become subject to export controls if they are intended in their entirely or in part for particular end uses including in connection with the development, production, handling, operation, maintenance, storage, detection, identification, or dissemination of chemical, biological or nuclear weapons or missiles capable of delivering such weapons
Council directive 2000/29/EC, 8 May 2000	On protective measures against introduction into the Community of organisms harmful to plants or plant products and their spread within the Community
Council directive 2001/83/EC, 6 November 2001	On the community code to medicinal products for human use
Council common position 2001/931CFSP, 21 December 2001	Establishes a list of suspected terrorists, defines terrorism to include those who manufacture, acquire, possess, transport, acquire, supply or use WMD. Requires Member States through police and judicial cooperation to assist each other in preventing and combating terrorist acts
Council directive 2001/82/EC, 6 November 2001	On the community code to veterinary medicinal products
Council directive 2001/18/EC, 12 March 2001	On the deliberate release into the environment of GMOs and repealing Council Directive 90/220/EEC. Establishes a regulatory framework for work on and release of GMOs. Provides risk assessment criteria and emergency response
Council regulation 2001/2432/EC, 20 November 2001	On a Community regime for the control of exports of dual-use items and technology
Council regulation 2002/880/EC, 27 May 2002	On a Community regime for the control of exports of dual-use items and technology
Council decision 2002/381/CFSP, 21 May 2002	Implements joint Action 1999/878/CFSP with a view to contributing to the EU cooperation program for non-proliferation and disarmament in the Russian Federation
Council common position 2002/976/CFSP, 12 December 2002	Most recent amendment of 2002/931/CFSP and its list, contained in the Annex. Also repeals the previous amendment 2002/847/CFSP
Council regulation 149/2003/EC, 27 January 2003	Amending and updating Regulation (EC) No 1334/2000 setting up a Community regime for the control of exports of dual-use items and technology
EC regulation 1504/2004	Includes exceptions from licensing and inclusions of technologies
Council regulation (EC) No 394/2006 of 27 February 2006	Amends and updates Regulation (EC) No 1334/2000 setting up a Community regime for the control of exports of dual-use items and technology
EC regulation number 450/2008	To establish a modern customs code
Council joint action 2008/307/CFSP	To support WHO activities in the area of laboratory biosafety and biosecurity in the framework of the EU Strategy against the proliferation of WMD
Commission directive 2009/41/EC	On the contained use of GMOs (this new Directive repeals Directive 90/219/EEC and its successive amendments Directive 94/51/EC, Directive 98/81/EC and Decision 2001/204/EC)
EC regulation number 428/2009	Sets up a Community regime for the control of exports, transfer, brokering and transit of dual-use items
EC communication COM(2009)273	On strengthening chemical, biological, radiological and nuclear security, which includes a proposal for an EU CBRN Action Plan

Based on National Implementation Database, EU section 2013

*GMOs* Genetically modified organisms, *GMMs* genetically modified microorganisms, *WMD* weapon of mass destruction, *CBRN* chemical biological radiological nuclear threats

Apart from legally binding agreements and legislative measures, biorisk management standards play supportive role, as well. Standards are kind of declaration of intention, code of conduct or guidance for good practice in particular area. Standards are based on existing international norms (WHO) and national/regional (EU) legislations. They are developed with the help of Non-Governmental Organizations dealing with topic issues directly (WHO, FAO, OIE, etc.) as well as authorizing institutions like European Committee for Standardization and International Organization for Standardization. Standards developed for biosafety and biosecurity management need to be applicable for governmental and private institutions and following, being a helpful tool for State Parties for more effectively implementation of the BTWC obligations.

List of the most important standards in biosafety and biosecurity fields is presented in Table 2.

**Table 2:** International standards related to biosafety and biosecurity sectors (some of these are national (CDC) or multinational (CWA))

Name	Objectives
Guidance on regulations for the transport of infectious substances 2013–2014, WHO/HSE/GCR/2012.12	Legislation, classification of infectious substances, preparation of shipments for transport, packaging, labeling and documentation requirements for infectious substances in category A and B, overpacks, reusing and shipping empty packaging, refrigeration, training, transport planning, procedures, incident reporting
Biosafety professional competence CEN CWA 16335:2011	Biological hazards, environment (working), laboratories, personnel, management, training, courses, education, examination (education), risk assessment, safety measures, occupational safety
UN regulations on transport of dangerous goods	These regulations also describe guidelines and regulations of transportation of dangerous pathogens
Laboratory biorisk management standard CWA 15793:2011	Biorisk management system: biorisk policy, planning (hazard identification, risk assessment, risk control), implementation and operation (responsibilities and authorities, personnel training, awareness and competence, consultation and communication, operational control, work practices, personnel protection, waste management, worker health program, infrastructure, transport of biological agents and toxin, personal security), emergency response and contingency plans, checking and corrective actions (data analysis, inventory monitoring and control, accident/incident investigation and preventive actions)
Responsible life sciences research for global health security, WHO guidance, 2010	WHA55.16 and the biorisk management framework for responsible life sciences research (research excellence, ethics, biosafety and laboratory biosecurity), self-assessment questionnaire
Bioterrorism incident pre-planning and response guide, second edition, INTERPOL, 2010	CBRNE threats, biological agents, overt and covert attacks, preparedness and operational response for bioterrorism
Natural ventilation for infection control in health-care settings, WHO guideline, 2009	Definition of ventilation, requirements for natural ventilation, describing the basic principles of design, construction, operation and maintenance for an effective natural ventilation system to control infection in health-care settings
Biosafety in microbiological and biomedical laboratories, CDC, 2009	Biological risk assessment (biological agents, laboratory procedures, work practices), biosafety (safety equipment, facility design and construction, animal facilities, clinical labs, shipment infectious materials), laboratory biosafety level criteria, vivarium biosafety level criteria, laboratory biosecurity, occupational health and immunoprophylaxis, containment for biohazards, decontamination, disinfection, agriculture pathogen biosafety
Green paper on bio-preparedness, EC, 2007	Key principles of bio-preparedness (prevention, protection, security issues related to biological research, public and animal health surveillance, response and recovery)
A code of conduct for biosecurity––reported by the Biosecurity Working Group, 2008	Biological weapons, dual use, threat analysis, life sciences and biological weapons, existing legislation, implementation and compliance with code of conduct
Biorisk management––laboratory biosecurity guidance, WHO/CDS/EPR, 2006	Laboratory biosecurity as a complement to laboratory biosafety, biorisk management, countering biorisks, laboratory biosecurity programme
Biological incident response and environmental sampling (European guideline on principles of field investigation), 2006	Field investigation (attack stages, biological agents, delivery devices, incident types), mission planning, arrival on scene, sampling strategy, safety (environmental, FIT), assessment and crime scene search
International Health Regulation, WHO, 2005	Foster global partnerships, national disease prevention, surveillance, control and response systems, public health security in travel and transport, strengthen WHO global alert and response systems and management of specific risks, obligations and procedures
Laboratory biosafety manual, fourth edition, WHO, 2004	Microbiological risk assessment, biosafety levels 1–4 (code of practice, lab design and facilities, lab equipment, health and medical surveillance, training, waste handling, chemical, fire, electrical, radiation and equipment safety), laboratory animal facilities levels 1–4, laboratory equipment, good microbiological techniques, biosafety and recombinant DNA technology, safety organization and training, safety checklist
Public health response to biological and chemical weapons, WHO guidance, 2001	Assessing the threat to public health, biological and chemical agents, public health preparedness for biological or chemical incidents and response, legal aspects, international sources of assistance
Guidelines for the collection of clinical specimens during field investigation of outbreaks, WHO/CDS/CSR/EDC/2000.4	Planning and processing for specimen collection, storage, packaging and transport of specimens

*CEN* European committee for standardization, *CDC* US centers for disease control and prevention, *CWA* communications workers of America, *CBRNE* chemical biological radiological nuclear explosive threats

## National Implementation

In accordance to BTWC, biosafety and biosecurity need to cover measures to account for and secure production, use, storage and transport of highly dangerous pathogens and toxins or activities connecting humans, plants or animals where infection or intoxication may pose a risk. Moreover, procedures relating licensing, safety and security measures for laboratories, laboratory containment measures and genetic engineering regulations need to be engaged, as well.

The national implementation of each State Party is essential to enforce the provisions of the BTWC, namely, prohibition and prevention of development, production, stockpiling, acquisition, retention, transfer or use of biological weapons by any person under their domestic jurisdiction. Furthermore, each country is obligated to implement additional measures into the current valid legislation to more preferably protect and prevent encouraging, inciting or assisting others.

National implementation of BTWC has been approved in most European countries so far, and determines the measures to account for and secure production, use and storage particularly dangerous pathogens or activities involving humans, plants or animals where infection may pose a risk (BTWC Legislation Database, VERTIC 2013).

## Penalization

The penalization measures related to biological safety and security are implemented in the national BTWC regulations. Countries without national implementation of BTWC regulate penalization issues fragmentarily by national Penal Codes, e.g., in section on penalization of crimes against international law (BTWC Legislation Database, VERTIC 2013).

## Protection of Employees Working with Biological Agents

Directive 2000/54/EC is the most important in protection of workers from risks related to exposure to biological agents at work.

National regulations on protection of employees working with biological agents have been implemented in most of the European countries (BTWC Legislation Database, VERTIC 2013). Austria regulate this issue by the Ordinance on protection of employees against hazards caused by biological agents, the Ordinance on work with GMOs in contained use (analogical ordinance applies in Switzerland), Law on the protection of employees, Regulation on biological work material and Regulation on safety of workplace (the last is similar to Ireland regulation). In Germany, apart from the Ordinance on safety and health protection at work involving biological agents, Technical Regulations for Biological Agents and Labour Protection Act are valid. The Royal Decree on protection of employees working with biological agents has been issued in Belgium. Comparable Decree on protection of employees’ health at work is valid in Czech Republic and France. In France, the Work Code that affects the use of biohazards as well as Order on health and safety signs at work has been additionally implemented. The objectives of Directive 2000/54/EC have been implemented also in Swedish, British and Norwegian national regulations. Provisions of Directive 2000/54/EC introduces into Polish law the Minister of Health of 22 April 2005 the harmful biological agents for health in the workplace and protection of the health of workers occupationally exposed to these factors. Simultaneously, the Labour Code requires the employer to evaluate and document the occupational risks associated with performed work, the use of the necessary preventive measures to reduce risk and to inform employees about the occupational risk that is associated with their daily work, and the principles of risk protection. Labour Codes in Belarus and Kazakhstan as well as the Labour Regulation Act in Macedonia regulate some aspects of above Directive. In Moldova, some obligations of Directive 2000/54/EC are implemented in Law on Biosecurity; nevertheless, it is mainly related to GMOs and GMO products.

## Genetic Engineering

Directive 2009/41/EC (repealing and replacing Directive 90/219/EEC and 98/81/EC) is the most important in contained use of genetically modified microorganisms (GMMs).

Austria, Ireland, Moldova, Germany, Norway, Poland, Portugal, Sweden and UK have implemented obligations of Directive 2009/41/EC in national regulations (BTWC Legislation Database, VERTIC 2013). In general, contained use of non-GM pathogenic microorganisms has not been taken into account. In Switzerland, the handling of GMOs is governed by the Gene Technology Law, while the handling of non‐GM pathogenic organisms is governed by the Federal Laws on Epidemics and Environmental Protection. The Ordinance on the contained use of organisms of 25 August 1999 (as of 23 November 1999) regulates the contained use of organisms, in particular genetically modified or pathogenic organisms.

## Waste Management

Waste management is regulated in almost all European countries as separate legal act (France, Belgium, the Netherlands, Switzerland, Germany, Poland, Turkey, Spain, Macedonia, Belarus, Bosnia and Herzegovina, Moldova). In Belgium and France, the Directive 98/81/EC is implemented into national legislation. In France, requirements of above Directive are completed by specific regulations on waste originating from medical care and dangerous waste in general, imposing rules for storage, for incineration and for collection by an approved company. The Netherlands implements Directive 2008/98/EC. Some countries implement both, GMOs and GMO products waste as well as contained material management as an annex to currently applicable law or code (Czech Republic, Sweden). The Albanian Law of Environmental Protection regulates the management of hazardous waste and dangerous matters. In Croatia are applicable both, the Regulation on Categories, Types and Classification of Waste with a Waste Catalogue and List of Hazardous Waste and Regulation on supervision of transboundary movement of waste (BTWC Legislation Database, VERTIC 2013).

## Handling of Laboratory Animals

National legislation concerning laboratory animal handling is neglected in almost all European countries. In France applies the Order of 19 April 1988 setting out conditions for the provision to accredited laboratories of animals used for purposes of experimental and scientific research, setting out conditions for issuing authorizations to conduct animal experiments, and setting out conditions for accreditation, fitting out and operation of animal experimentation establishments. Some elements including isolation or removal of sick animals, disinfection, safety lodge of the animal carcasses, rendering and other wastes, in an approved place, safety lodge or sanitary treatment of the excrement as well as elimination of transmission vectors of the contagious diseases and safety handling of animal cadavers are regulated by national legislations concerning veterinary service and inspectorate (e.g., Albania). Other elements are regulated in Law on health and well-being of animals (e.g., the Netherlands) (BTWC Legislation Database, VERTIC 2013).

## Reporting of Accidents

In general, reporting of incidents and accidents in laboratories is included in national ordinances, acts and regulations (Denmark, Switzerland, Belgium, Portugal, United Kingdom, Spain, Poland, Serbia, Turkey, Ukraine, Macedonia, Kazakhstan). In some countries, this aspect is specifically related to GMO and GMO products, e.g., Austrian Ordinance on Work with GMOs in Contained Use regulates safety aspects and the measures to be taken in case of accidents. A Czech Republic Law Act on GMO Products covers accident reporting in Article 20 and 21, concerning, respectively, emergency response plan and measures taken in case of an accident. Nevertheless, no uniform mechanism to report on incidents/accidents in European countries has been established. Some legal acts mention about necessity of it, but do not provide detailed information how to do it or provide only overall information (BTWC Legislation Database, VERTIC 2013).

## Laboratory Measures Related to Biosafety and Biosecurity

Legal obligation on Standard Operational Procedures is applicable in many countries in relation to biological safety and security. In general, procedures and Good Laboratory Practice issues are within responsibility of directors and supervisors of the laboratories as occupational safety and health rules.

## Transportation of Biological Agents

National measures to account for and secure transport of particularly dangerous pathogens or activities involving humans, plants or animals where infection may pose a risk have been analyzed. Most of the European countries have applicable regulation concerning transport of dangerous goods (Austria, the Netherlands, Germany, Bulgaria, Denmark, Estonia, Finland, France, Spain, Latvia, Macedonia, Moldova, Liechtenstein, Norway, Poland, Portugal, Russian Federation, Romania, Serbia, Slovak Republic, Turkey, Ukraine, Kazakhstan, Iceland, Lithuania, Montenegro, Andorra). Some countries have also implemented Law on transportation of hazardous goods (e.g., Switzerland) or both (e.g., UK, Czech Republic) (BTWC Legislation Database, VERTIC 2013).

## Dual-Use Technology

National Act implemented Council Regulation (EC) No 428/2009 setting up a Community regime for the control of exports, transfer, brokering and transit of dual-use items which is directly applicable in all EU countries (Austria, Belgium, Bulgaria, Cyprus, Czech Republic, Denmark, Estonia, Finland, France, Germany, Greece, Hungary, Ireland, Italy, Latvia, Lithuania, Luxemburg, Malta, the Netherlands, Poland, Portugal, Romania, Slovakia, Slovenia, Spain, Sweden, UK) (BTWC Legislation Database, VERTIC 2013). Candidate countries for EU membership are Turkey, Croatia and the Former Yugoslav Republic of Macedonia.

## Biosecurity Measures

Only three European countries have a Law on Biosecurity (Sweden, Moldova, Turkey), but in general it is focused on GMOs and GMO products. The physical containment of laboratories is dedicated particularly to GMOs. Law on national security of Romania covers overall issues. Executive Order on securing specific biological substances, delivery systems and related material is applicable in Denmark. Legal requirements to impose secure storage and use of dangerous pathogens and toxins are listed in Schedule 5 of Part 7 of the Antiterrorism, Crime and Security Act 2001 and the Security of Pathogens and Toxins (Exceptions to Dangerous Substances Regulations 2002) in UK (BTWC Legislation Database, VERTIC 2013).

No EU level legislation exists that has been specifically developed to address the protection of biological agents in the laboratory from loss or misuse, thus, biosecurity aspect is in principle neglected. Nevertheless, due to many synergies between biosafety and biosecurity, the EU Directives developed to protect workers from exposure to biological agents or GMMs address most of the issues related to laboratory biosecurity. Overall, competences and responsibilities of persons responsible for biosafety and biosecurity are fragmentarily defined. Licensing staffs to authorized access to the laboratory is neglected in most countries. Many institutions implement some biosecurity controls, but these are often focused on physical security. Less attention is focused on information security or organizational security issues, despite the fact that internal threats from individuals with authorized access to the laboratory must be recognized.

## Bioethics

In most of the European countries, national code of ethics for scientific research or codes of bioethics is applied, e.g., Belgium, Czech Republic, the Netherlands, Ireland, Latvia, Germany, UK, Switzerland, Italy. In Italy, additionally the Conduct for Biosecurity has been implemented in 2010. Other countries use manuals or guides, e.g., Good Practice for Scientific Research and Good Manners in Science issued by the Polish Academy of Science.

## Conclusion

In general, most of the biosafety and biosecurity aspects are covered independently in national laws, procedures and on technical and physical measures related to human, plant and animal pathogens. Based on the national legislation review, risk management is better documented for GMOs than for native pathogens. There is lack of legislative unification in GMOs and pathogenic agents. Usually, the GMOs regulation covers biosafety and biosecurity issues only in relation to GMOs and GMO products.

Still applicable becomes a need for global unification of national regulations in biosafety and biosecurity fields (Stavskiy et al. 2003).
